# Real Time Electronic Feedback for Improved Acoustic Trapping of Micron-Scale Particles

**DOI:** 10.3390/mi10070489

**Published:** 2019-07-21

**Authors:** Charles P. Clark, Vahid Farmehini, Liam Spiers, M. Shane Woolf, Nathan S. Swami, James P. Landers

**Affiliations:** 1 Department of Chemistry, University of Virginia, Charlottesville, VA 22903, USA; 2 Department of Electrical and Computer Engineering, University of Virginia, Charlottesville, VA 22903, USA; 3 Departments of Mechanical Engineering and Pathology, University of Virginia, Charlottesville, VA 22903, USA

**Keywords:** acoustic differential extraction, feedback, frequency

## Abstract

Acoustic differential extraction has been previously reported as a viable alternative to the repetitive manual pipetting and centrifugation steps for isolating sperm cells from female epithelial cells in sexual assault sample evidence. However, the efficiency of sperm cell isolation can be compromised in samples containing an extremely large number of epithelial cells. When highly concentrated samples are lysed, changes to the physicochemical nature of the medium surrounding the cells impacts the acoustic frequency needed for optimal trapping. Previous work has demonstrated successful, automated adjustment of acoustic frequency to account for changes in temperature and buffer properties in various samples. Here we show that, during acoustic trapping, real-time monitoring of voltage measurements across the piezoelectric transducer correlates with sample-dependent changes in the medium. This is achieved with a wideband peak detector circuit, which identifies the resonant frequency with minimal disruption to the applied voltage. We further demonstrate that immediate, corresponding adjustments to acoustic trapping frequency provides retention of sperm cells from high epithelial cell-containing mock sexual assault samples.

## 1. Introduction

The forensic community is hampered by a significant backlog of evidence that requires DNA analysis, with a sizable subset of this consisting of sexual assault samples [1,2,3]. This stockpile of evidence awaiting testing exceeds 100,000 samples in the United States, with many laboratories requiring a minimum of four months for analysis of each sample [4,5]. A contributing factor to this backlog is the current methodology used for obtaining DNA identification from sexual assault evidence. DNA analysis to generate a short tandem repeat (STR) profile from the sperm cells in sexual assault evidence involves sample extraction, amplification of target DNA, and electrophoretic separation of DNA fragments to conclusively identify an individual. However, before DNA analysis can be completed, sperm cells must be isolated from the rest of the genetic material in the sample. To achieve this, each sample undergoes differential extraction, a technique that relies on multiple centrifugation and washing steps to pellet sperm cells after female epithelial cells (E-cells) are lysed, allowing for extraction of only male DNA [6]. This widely accepted approach is effective, but is labor-intensive, requires a highly trained analyst and can take multiple hours per sample [7]. Hence, there is a significant need for a faster, more automated technique to isolate sperm cells from sexual assault samples. By removing the need for a skilled analyst to perform manual separation steps, cell loss is circumvented, more efficient processing of samples results, which ultimately leads to faster resolution of investigations.

Acoustic differential extraction (ADE) is an attractive alternative to conventional differential extraction for sexual assault samples. As described in our previous work [8,9,10], ADE offers an automated approach for separating sperm cells from the E-cell lysate and other cellular debris, and can be incorporated with existing chemical protocols in forensic laboratories. The ADE approach generates a standing acoustic wave within a microfluidic chamber, using sound waves to capture and purify sperm cells. This is made possible by performing a differential lysis, which ruptures all non-sperm cells in a sexual assault sample, leaving sperm as the largest (and only intact) particles in solution. When subjected to a standing acoustic wave, those intact sperm cells will experience an acoustic radiation force, and can be held in place under moderate flow conditions while all other cellular debris and free DNA is washed away. The ADE technique works effectively with mock sexual assault samples [10], and compares favorably to conventional DE for authentic sexual assault samples [11]. The crucial component of ADE is the acoustic radiation force, which causes particles to aggregate at low-pressure nodes due to their acoustic contrast with the surrounding medium, with larger particles experiencing a stronger primary acoustic radiation force [12]. As shown in Equation (1), the primary radiation force (*F*_r_) is dependent on the acoustic pressure amplitude (p_0_), the volume of the particle (V_c_), the compressibility of the fluid (β_w_), the wavelength of applied sound (λ), the wavenumber (*k*), the distance from a pressure node (*x*), and the acoustic contrast factor (ϕ). The acoustic contrast factor itself can be expanded (Equation (2)) and is a function of the compressibility and density of both the particle (ρ_c_ and β_c_) and the surrounding fluid (ρ_w_ and β_w_).

(1) $$F_{r}=-\left(\frac{\pi p_{0}^{2}V_{c}\beta_{w}}{2\lambda }\right)\cdot \phi \left(\beta ,\rho \right)\sin\left(2kx\right)$$

(2) $$\phi \left(\beta ,\rho \right)=\frac{5\rho_{c}-2\rho_{w}}{2\rho_{c}+\rho_{w}}-\frac{\beta_{c}}{\beta_{w}}$$

A factor that is not included in these equations, but which is crucial for understanding the effect described, is the sound velocity. Changes to density and compressibility of a fluid will alter the speed of sound in that liquid, as shown in Equation (3) (*v* = speed of sound, *K* = compressibility, ρ = density). Changes to sound velocity, in turn, will alter the wavelength generated by an applied frequency, as seen in Equation (4) (c = speed of sound, λ = wavelength, *f* = frequency). Thus, shifts in fluid density or compressibility, which impact sound velocity, will necessitate a corresponding shift in applied frequency to generate a resonant acoustic wave.

(3) $$v={\left(K\rho \right)}^{1/2}$$

(4) $$\lambda =\frac{c}{f}$$

In theory, this suggests that significant changes to the compressibility or density of the liquid medium would result in a similar shift in the required frequency, essentially changing the resonance condition. What makes these parameters crucial for changing the resonance condition is that they are variable with each sample, unlike the particle volume, compressibility, or density, which remain roughly constant for sperm cells across human males [13]. The fluid density and compressibility may be altered by drastic changes in cell concentration before and after lysis, in turn causing a shift in the frequency of sound required to generate a resonant acoustic wave. The resonance condition of an acoustic oscillator is dependent on the sound velocity, frequency, and wavelength, as well as the cavity dimensions. Changes to any of these parameters will impact the resonance condition, which in turn affects the pressure amplitude of the standing wave. The degree of change to the pressure amplitude depends on the Q-factor of the resonator, a measure of a system’s oscillation at resonance compared to its bandwidth [14]. For a high-Q material, like the glass resonator cavity in this application, the amplitude–frequency relationship has a narrow range, where deviation from the resonant frequency will result in drastic loss of acoustic pressure amplitude [15]. That loss manifests as weaker acoustic trapping, and failure to retain particles. Thus, changes in resonance condition due to alteration in fluid compressibility or density, must be addressed by shifting the applied frequency.

The fluidic properties of each sample can vary with different pieces of sexual assault evidence, which can subsequently impact acoustic trapping performance. This effect was made clear during testing of the ADE prototype in an external forensic laboratory, where sperm cells were easily captured in some cases, but acoustic trapping suffered with some other samples. Some sexual assault samples will contain extremely high concentrations of E-cells (<50,000 cells), which upon differential lysis will significantly change the physical properties of the sample media. We hypothesized that, with potentially hundreds of thousands of E-cells present in a single sample, lysis alters the compressibility and density of the sample, thereby impacting the resonance condition, which is to say the necessary wavelength and frequency of sound required will shift. This phenomenon has been described more generally in the literature, with multiple studies showing that when lysis occurs, the cellular content perfuses into the surrounding liquid and causes an increase in viscosity [16,17,18,19]. There is an empirical dependence between viscosity, density, and compressibility of a liquid [20,21], and more specifically to acoustic trapping applications, groups have shown that changes in viscosity of the surrounding fluid impacts the acoustic radiation force experienced by particles in a standing wave [22,23]. With a proper understanding of the relationship between these parameters, and the knowledge that cellular lysis and variation among samples will impact fluid properties, we assume that significant changes in density and compressibility due to sample characteristics will adversely affect the ability to trap sperm cells due to a shift in optimal resonant frequency.

Sexual assault samples can vary dramatically in their makeup, containing anywhere from thousands to hundreds of thousands of cells. This wide range of possible samples means that the optimal acoustic trapping frequency will change on a sample-to-sample basis, which could have a devastating impact: It has been shown that a deviation of as little as 0.05 MHz from the ideal trapping frequency can result in a 10-fold loss of trapping efficiency [24]. In terms of our application, which uses between 7–8 MHz, this change is only a shift of less than 1% of the applied frequency. As it impacts ADE, this would mean that if the applied acoustic frequency is not precisely regulated for each sample, loss of trapping efficiency would occur and the majority of the sperm cells could be lost. An elegant solution is required to accommodate the bandwidth of samples encountered, specifically, adjust the acoustic trapping parameters to the character of each sample. Inspired by pioneering work from the Nilsson/Laurell group at Lund University [24], we are developing a feedback system that can automatically adjust the applied acoustic frequency through rapid electronic measurements. Previous literature suggested that impedance can be used to predict the optimal trapping frequency by monitoring output voltage [25,26,27], and a similar approach was adapted by the Lund group. Their work showed that, by calculating a root mean square power spectrum from the measured impedance and output impedance of their function generator, the optimal trapping frequency could be updated continuously during acoustic trapping. This allowed for automated adjustment of the trapping frequency as the surrounding medium was alternated between two very different solutions—a wash buffer and human plasma. The change in media led to a shift of 0.03 MHz in optimal trapping frequency, but the frequency tracking system successfully adjusted and continued to effectively trap 12 μm diameter particles. We postulated that this approach might be effective for acoustic differential extraction. During the ADE protocol, a variety of solutions traverse the trap zone—water, sexual assault sample cell lysate, bead solution, and wash buffer. Each of these constitutes a significant change in the acoustic trapping environment. Hence, a feedback system for sensing those changes, and subsequently adjusting the optimal trapping frequency, is needed to prevent unacceptable loss of sperm cells during the trapping process.

The main aim of this work was to modify the hardware in the ADE prototype [10] to facilitate real-time feedback that reflects a change in the medium conditions during trapping, ultimately enabling efficient acoustic trapping of sperm cells from sexual assault samples. By measuring the voltage drop across the piezoelectric transducer during the acoustic trapping event, we sought to identify reproducible trends that can inform real-time changes. From these relationships between output voltage and optimal trapping frequency, applied parameters could be adjusted and improved during the ADE process. The specific parameter, which is most crucial to precisely tune is the applied acoustic frequency, which is unique for each test depending on both the microchannel height and the liquid properties of that specific ample. Our hypothesis was that, by monitoring the voltage output from the piezoelectric transducer, we would be able to rapidly identify the optimal trapping frequency as it changes during sample trapping due to differences in liquid properties between each solution. This information could then be used to adjust the applied frequency to avoid suboptimal trapping and, hence, prevent loss of sperm cells. To summarize the big picture, our previous work implemented visual measurements of bead trapping to account for chip-to-chip variation and tune the applied frequency. This work seeks to account for sample-to-sample variation, and adjust in real-time based on rapid voltage output measurements.

## 2. Materials and Methods 

### 2.1. Instrumentation for Real Time Electronic Feedback

The acoustic differential extraction prototype has been described in depth by our previous publications [9,10]. Precise fluidic control is achieved via two solenoid valves (LabSmith AV201, LabSmith Inc. Livermore, CA, USA) and three syringe pumps (LabSmith SPS01, LabSmith Inc. Livermore, CA, USA), which interface with the microchip pneumatically, preventing any liquid contact or contamination of the hardware. The electronic components of the instrument include a waveform generator (Hantek DDS-3X25, Hantek, Shandong, China), oscilloscope (Hantek 6052BE, Hantek, Shandong, China), and home-built amplifier, which provide control over the applied acoustic frequency. The same previously reported instrument was used for this study, however there were significant additions to the electronic domain, namely the incorporation of a custom printed circuit board (PCB) that measured the voltage output of the piezoelectric transducer during an acoustic trapping test. 

In the classic peak detector, used in previous works (24, ESI), the holding capacitor is charged with a current (I_c_), which is highly dependent of capacitor voltage (V_c_). This current decreases as the capacitor voltage increases. The capacitor voltage should ideally become equal to V_a_ that is the amplitude of attenuated signal after the voltage divider unit. However, when the capacitor is charging toward the desired level of V_a_, the charging current is not high like when the capacitor is empty. This reduced current in higher frequencies may be unable to completely charge the capacitor, therefore the amount of error (relative difference between V_c_ and V_a_) may increase accordingly. The trapping frequency of biological samples in our experiments is around 7–8 MHz where the classic peak detector does not provide the sufficient accuracy (>5% error) that is required for discerning subtle variations of signal around resonant frequency.

**Figure 1:**
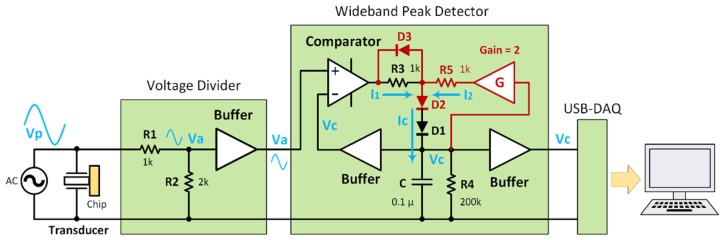
The simplified schematics of the wideband peak detector and the key voltage points.

As a solution to this limitation, we used an upgraded design for the classic peak detector, called the wideband peak detector. The details of operation have been fully explained in an online publication [28], and a simplified schematic of the wideband peak detector is included below. Figure 1 shows the simplified schematics of the circuit used for precise measurement of the AC voltage amplitude across the piezo transducer. The incoming voltage (*V_p_* ≈ 18 Vpp) was attenuated by the factor of three through the voltage divider unit. The maximum amplitude at the output of this unit (V_a_) was around 6 Vpp that lies within the allowable input range of the peak detector unit. The DC voltage corresponding to the peak amplitude (V_c_) was stored in the holding capacitor (C). A fast comparator (Ad8561, Analog Devices, MA) continuously compared the already held value of the peak with the input AC voltage (V_a_). Whenever the present value of input on the positive input of the comparator went higher than the DC voltage on its negative input (V_c_), the comparator output swung high (+5V). It gave rise to a charging current (I_c_) through R3 and the diode (D) increases the capacitor voltage. When the input AC voltage (V_a_) went below the capacitor voltage (V_c_), the comparator output went down (≈ 0 *V*) and the capacitor retained its voltage due to the unidirectional behavior of diode. In steady state, the capacitor holds the peak level of the input signal. The red-colored circuitry in Figure 1 was added to convert the classic peak detector to the wideband peak detector. In short, the amplifier (Gain = G) acts as an additional current source (I_2_) for the capacitor in a way that the sum of I_1_ and I_2_ is relatively a constant value equal to I_c_, independent of the capacitor voltage (V_c_) as confirmed by Equation (5). By choosing appropriate values for R_3_, R_5_, and G, the second term in Equation (5) becomes zero and I_c_ can be expressed as Equation (6), independent of V_c_.

(5) $$I_{c}=I_{1}+I_{2}=\left(\frac{5-2V_{D}}{R_{3}}+\frac{-2V_{D}}{R_{5}}\right)+V_{c}\left(\frac{-1}{R_{3}}+\frac{G-1}{R_{5}}\right)$$

(6) $$I_{c}=\frac{5-4V_{D}}{R_{3}}$$

The peak detector should be able to track the variation of input amplitude with an acceptable time lag (<0.1 s), therefore the resistor R4 was added to deplete the capacitor in case the input voltage drops to lower values. However, R4 should be large enough to avoid voltage ripple on V_c_. The DC voltage buffers in the peak detector unit were implemented with very high input impedance operational amplifiers (OPA4170, Texas Instruments, Dallas, TX, USA) to minimize the charge loss in the capacitor. However, a wide bandwidth operational amplifier (LM6171, Texas Instruments, Dallas, TX, USA) used for the voltage buffer in the voltage divider unit to preserve the signal amplitude at frequencies around 8 MHz. The wideband peak detector collected voltage data and transfers it via a data acquisition card (National Instruments, Austin, TX, USA) to an external laptop. The voltage data was plotted in real time through LabView software, which displays the output voltage as a function of time. This information was then used by the operator to select a new acoustic frequency during sample trapping.

### 2.2. Chemical Protocol for Cellular Lysis and Mock Samples

Buccal swabs were collected from healthy donors to be used in this study. The cotton swabs were reconstituted in 600 μL H_2_O and agitated for 60 s to remove cells from the substrate. Cellular concentration was determined via hemocytometry with the green fluorescent nucleic acid stain SYTO-11. Sperm cells were collected from anonymous healthy donors, and cell concentration also determined via hemocytometry. E-cells were lysed using the prepGEM reagent (ZyGEM NZ Ltd, Hamilton, New Zealand) and incubated according to manufacturer instructions.

## 3. Results

### 3.1. Microchip Design for Acoustic Capture of Sperm Cells

**Figure 2:**
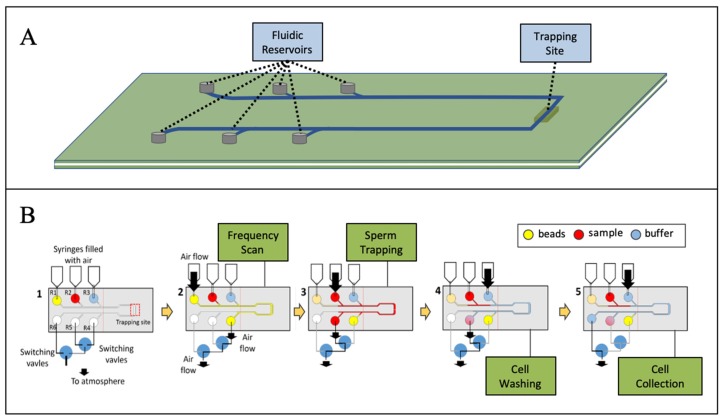
Acoustic differential extraction (ADE) microchip and workflow. (**A**) The multilayer chip composed of glass, polydimethylsiloxane (PDMS), and poly(methyl)methacrylate (PMMA) contains six reservoirs and one acoustic trapping site. (**B**) Workflow for the trapping, washing, and capture of sperm cells from a sexual assault sample. The “upstream” reservoirs 1, 2, and 3 contain fluorescent beads, sample, and wash buffer, respectively. The “downstream” reservoirs 4, 5, and 6 are for the collection of the sperm fraction, non-sperm fraction, and waste fraction, respectively.

A glass-PDMS-glass microchip was used for the resonator domain, with a poly(methyl)methacrylate (PMMA) layer affixed for sample reservoirs and downstream collection chambers. Microfluidic architecture was designed in computer-assisted drawing software, and features ablated using CO_2_ laser-etching. Layers were adhered through solvent bonding, plasma oxidation, and pressure-sensitive adhesive. In the schematic shown in Figure 2, reservoirs 1, 2, and 3 were filled with fluorescent beads, the sexual assault sample lysate, and a wash buffer, respectively. The first step for sample processing is to flow fluorescent beads from reservoir 1 through the trap site, while scanning through eight different frequencies covering 0.14 MHz. By using both electronic measurements and visual camera-based monitoring of the trap site, the optimal trapping frequency can be determined for that specific microchip. The optimal trapping frequency is specific to the height of the resonant chamber and, thus, will shift slightly due to variation in material thickness between each microchip. Samples containing lysed E-cells and intact sperm cells were mobilized (pneumatically) from reservoir 2 through the trap site, while the previously defined optimal trapping frequency was applied. During the trapping process, the applied frequency was shifted up by 0.01 MHz every 2 s, and real-time voltage data from the piezoelectric transducer used to maintain the optimal trapping frequency regardless of any shift in sample composition. Finally, the piezo frequency was maintained at the previously determined optimal frequency (typically between 7.4–7.8 MHz) to retain all trapped cells in the trap zone, while wash buffer flowed from reservoir 3 to remove any residual free DNA or cellular debris from the pellet.

### 3.2. Effect of Increased E-cell Concentration on Acoustic Trapping Efficiency

**Figure 3:**
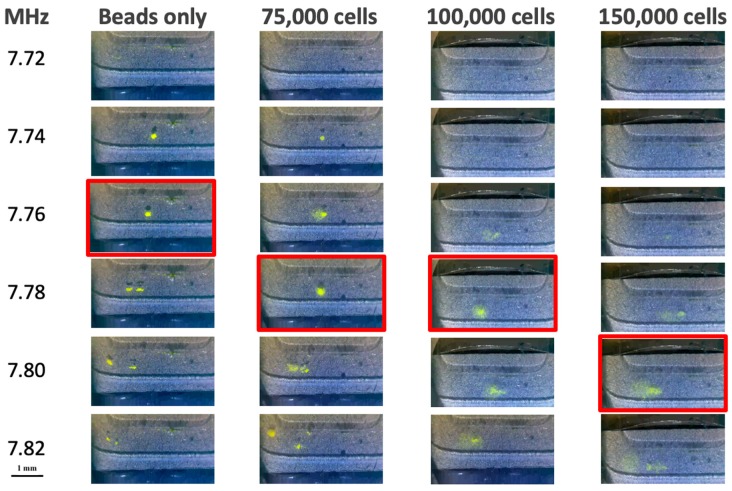
Shift in optimal trapping frequency with increasing epithelial cell concentration. Over a range of six applied frequencies (*y*-axis) different acoustic trapping was achieved, visualized by the aggregation of yellow fluorescent beads. In samples with increasing concentration of lysed epithelial cells (*x*-axis) there was a clear increase in the optimal trapping frequency.

In order to experimentally confirm our observed (during prototype development) and anecdotal (from forensic labs who evaluated the prototype) findings, a study was conducted to measure the impact of lysed E-cell concentration on acoustic trapping frequency. By trapping fluorescent beads in solutions of varying E-cell concentration, using the same microfluidic chip and hardware, any change in the optimal trapping frequency should theoretically be attributed to the E-cell concentration. Figure 3 shows the visual aggregation of fluorescent beads in the acoustic trap site, which is used to determine optimal trapping frequency. Solutions were tested with beads suspended either in water, lysate from 75,000 cells, lysate from 100,000 cells, or lysate from 150,000 female epithelial cells. Each solution was exposed to six different applied frequencies. Optimal trapping frequency was determined by visual identification of the largest single aggregate of fluorescent particles, as determined by an algorithm that measured the number of yellow pixels in each frame. The data shows that this optimal frequency is 7.76 MHz for beads in water, but shifts to 7.78 MHz for 75,000 and 100,000 cells, and up to 7.80 MHz for 150,000 cells. This may seem like a miniscule change in frequency, but note that if 7.76 MHz was used, the vast majority of the cells would fail to trap and, hence, be lost from 100,000 and 150,000 cell samples. This simple experiment clearly demonstrates that the sample E-cell concentration dramatically influences the optimal trapping frequency, and that when an overwhelming number of E-cells are present, trapping could be obliterated. Given the sample-to-sample differences common in forensic evidence, it became clear that a feedback system was required, one that could adjust the applied frequency in real-time to match the sample constituency in terms of E-cell concentration. This forces a new standard, one that uses beads in water to define the chip-specific frequency (correlating to chamber height [9]), but in addition, uses a sample-specific adjustment in real-time to assure that optimal sperm cell capture ensues. Stated differently, the bead-determined initial trapping frequency defines the general frequency region for trapping under ideal conditions, while sample-specific voltage measurements determine the real-time trapping frequency for optimal sperm cell retention.

### 3.3. Output Voltage Measurements Can Identify Optimal Trapping Frequency

During acoustic trapping, the resonance frequency occurs when the piezoelectric transducer most efficiently converts input electrical energy into mechanical energy [29]. As the piezo approaches its resonant frequency there was less resistance to vibration, meaning that electrical impedance would be at a minimum when resonance was achieved. This relationship is how Nilsson et al. successfully determined the optimal frequency for their acoustic trapping system, using impedance measurements to identify when resistance to vibration was at a minimum. Our approach uses this same principle, seeking to identify the piezo’s resonance by determining at what frequency the minimum opposition to vibration occurs. However, instead of using an impedance analyzer, we directly measure the ‘output voltage’ of the piezoelectric transducer. Impedance itself is defined as the opposition a circuit presents to a current when voltage is applied, and Ohm’s Law (for AC) states that V = IZ (V = voltage, I = current, Z = impedance) [30,31]. Therefore, by monitoring the output voltage of our acoustic trapping circuit, any changes in the output voltage represents a change in impedance and, thus, changes in the resistance to vibration of the piezo. Although the piezo is bonded to a glass coupling layer, the high-Q value of glass allows for efficient accumulation of acoustic energy [32], so any detectable changes in output voltage can be accurately attributed to piezo resonance rather than thermal effects or energy loss from the glass layer.

**Figure 4:**
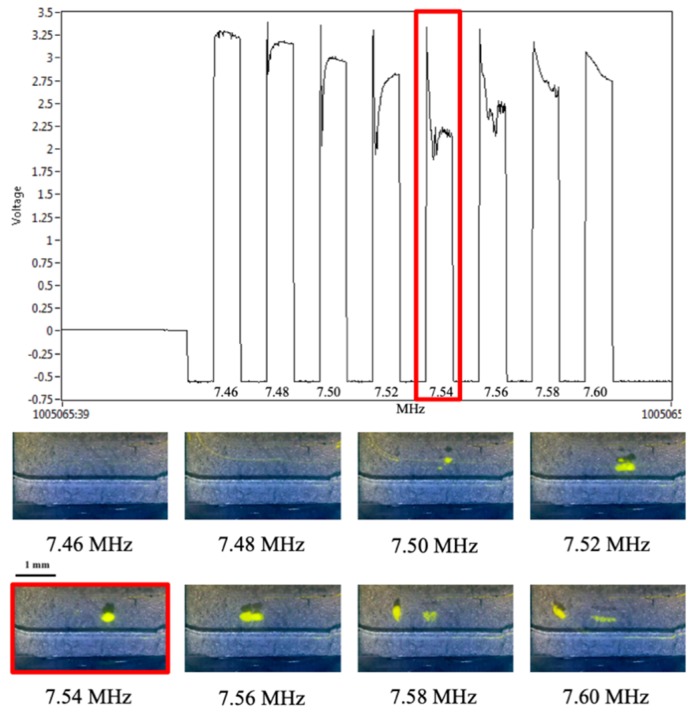
Voltage response to acoustic trapping. Electronic measurements of the piezo during acoustic trapping can be matched to visual data gathered via video monitoring of the trapping event. The lowest voltage output occurred at an applied frequency of 7.54 MHz, matching the frequency at which the optimal visual trapping of fluorescent beads was observed.

This principle was demonstrated by trapping fluorescent beads in a glass microchip at eight different acoustic frequencies. By simultaneously monitoring the voltage output of the piezo at each frequency, and visually monitoring aggregation of the beads, we can determine when optimal trapping occurs and decipher whether there is any concurrent trend in voltage out. Figure 4 shows the results of this experiment, plotting the output voltage (y-axis) against the applied frequency (x-axis), overlayed with still frames from video monitoring of the trapping at each frequency. Two conclusions can be drawn from this data; first, a clear minimum in the output voltage can be identified at 7.54 MHz. Second, this correlates with the strongest aggregation of fluorescent beads (identified as a single, dense aggregate in the middle of the channel) at 7.54 MHz. This suggests that the optimal trapping frequency can be determined from measuring the output voltage, rather than relying on visual monitoring of the bead aggregate size. The implication of this result is that rapid voltage measurements carried out during sample testing should be effective in identifying the optimal trapping frequency. Given this correlation between these two parameters, it is reasonable to expect that real-time adjustments could be made to the applied frequency to retain the maximum number of sperm cells despite changes in the medium (liquid) environment.

### 3.4. Shift in Optimal Trapping Frequency with Changes to Fluidic Properties

**Figure 5:**
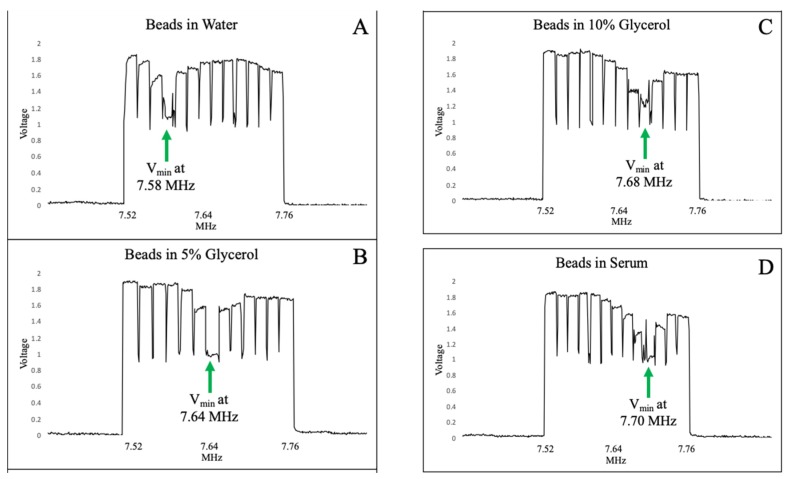
Shift in voltage minimum with changing viscosity. (**A**) A clear minimum in voltage output was observed at 7.58 MHz, indicating the optimal trapping frequency. (**B**) When flow was switched to 5% glycerol, the optimal trapping frequency shifted up to 7.64 MHz. (**C**) In 10% glycerol, the optimal trapping frequency shifted to 7.68 MHz. (**D**) In human serum, the minimum output voltage was 7.70, most similar to 10% glycerol.

If the problem at the core of ADE inefficiency with a broad range of sexual assault samples was differences in liquid properties as the number of epithelial cells varies, it was imperative to understand the relationship between those differences and output voltage. The use of a pristine solution of glycerol varying in concentration to alter viscosity was the simplest approach that evaluates changes in output voltage during rapidly changing fluidic conditions. An ADE microchip was loaded with three solutions: (A) Fluorescent beads in water (reservoir 1), (B) fluorescent beads in 5% glycerol (reservoir 2), and (C) fluorescent beads in 10% glycerol (reservoir 3). By first conducting a frequency scan from 7.52 MHz to 7.76 MHz with the water solution, then rapidly switching flow to the 5% and 10% glycerol solutions, respectively, abrupt changes in optimal trapping frequency were measured solely via voltage output from the piezo. Figure 5A shows the voltage output data during a frequency scan of fluorescent beads in water where a clear minimum was observed at 7.58 MHz, indicative of the optimal trapping frequency. However, when a 5% glycerol solution flows through the trapping zone, the minimum output voltage shifts to 7.64 MHz (Figure 5B). Similarly, when this is followed by trapping beads in 10% glycerol, the minimum output voltage (indicating optimal trapping frequency) increased to 7.68 MHz (Figure 5C). This confirms the hypothesis that changing viscosity of the sample will directly impact acoustic trapping ability. Samples with higher percent glycerol were tested, further shifting the minimum output voltage. At a 20% glycerol solution some focusing of particles was observed, and at 30% glycerol the limit of acoustic trapping was reached, with no particle aggregation.

### 3.5. Application of Real Time Feedback to a Mock Sexual Assault Sample

**Figure 6:**
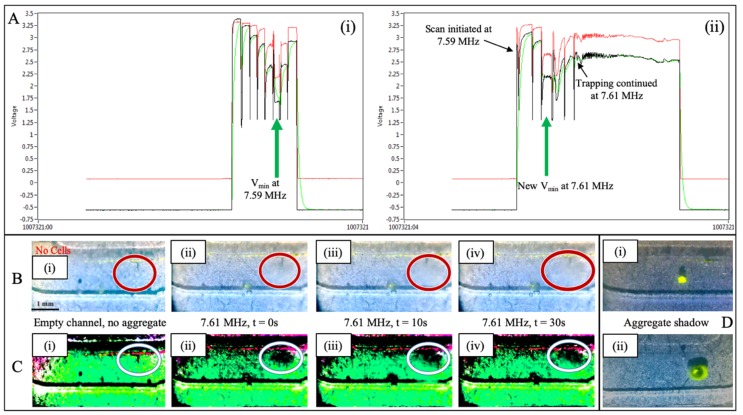
Real-time feedback during mock sample trapping. (**Ai**) Trapping fluorescent beads in water across eight frequencies shows a clear voltage minimum at 7.59 MHz. (**Aii**) While trapping the mock sexual assault sample, the applied frequency was adjusted by 0.01 MHz each step. A local minimum in average voltage was observed at 7.61 MHz, so that frequency was applied for the remainder of the trapping. (**Bi–iv**) Visual monitoring of the trap site over 30 s. At the conclusion of the trapping event, a large aggregate of sperm cells was captured. (**Ci–iv**) Color adjusted images from trapping site. Image processing allows for sperm aggregate to be clearly identified. (**D**) Images of beads (**i**) and beads with sperm cells (**ii**) being trapped. The shadow above and behind the aggregate is clearly visible due to the angle of lighting above the microchip.

Having demonstrated the relationship between voltage output of the piezo and optimal trapping frequency, mock sexual assault samples were prepared to test the feedback system. A sample containing 290,000 total cells, with a cell ratio of 5:1 female E-cells:male sperm cells, was prepared with a standard differential lysis procedure [6], and the resultant cell lysate exposed to acoustic differential extraction. Using the previously described feedback system deemed “ResFinder” a rapid scan of eight different frequencies was conducted using a ‘scanning solution’ consisting of fluorescent beads suspended in water. With the purpose of identifying the optimal trapping frequency for this specific microchip, the scanning process was executed in <20 s, and the data shown in Figure 6Ai clearly indicates that a minimum in voltage output occurred at 7.59 MHz—the optimal trapping frequency for that ADE chip. With a frequency ‘starting point’ of 7.59 MHz, the mock sample was then flowed through the trapping site and with the piezo activated. Figure 6Aii shows the data collected during sample trapping, where the applied frequency was increased by 0.01 MHz while monitoring the voltage output of the piezo. When a minimum voltage output was identified as 7.61 MHz, scanning was terminated and 7.61 MHz was utilized for the remainder of the sample trapping. This resulted in the capture of a large aggregate of sperm cells over the 30 s trapping event, which can be visualized by the increasing size of the sperm cell clump seen in Figure 6, panels B and C. Panel B shows the brightfield microscopic view of the trapping site, as sperm cells are collected in a flow stream over a period of 30 s. The part of the trap site highlighted by the red ellipse shows no cells in Figure 6Bi while the ‘blurred’ area in Figure 6Bii–iv represents the growing aggregate of cells. While a trained eye can easily discern the cell aggregate in Panel B, Panel C shows the same images after ‘color adjustment’. Color manipulation and image analysis are increasingly commonplace, and can be driven by simple smartphone ‘apps’ or by open source image processing software [33,34]. RGB is a common color space for image capture and for reporting image analysis results [35,36]. HSB (hue/saturation/brightness) color space transforms raw RGB values into a more perceptual color space, and has been routinely employed in our lab for qualitative and quantitative purposes; this is particularly useful for the investigation of colorimetric chemical reactions [37,38]. However, manipulation of color in RGB and HSB color spaces can be cumbersome and unintuitive. Here we exploit L*a*b* color space, which more closely approximates human vision and, as such, can be more intuitive, thus, easing the color manipulation process. In fact, L*a*b* is one of the more powerful and instinctive photo editing color modes used, for example, in Adobe Photoshop. Briefly, two key color adjustments were applied in L*a*b* color space using the ImageJ/Fiji 3D Color Inspector plugin (v2) [39,40,41,42]. Color contrast was boosted x14.09 and the color was rotated −90°. This color manipulation approach permits better visualization of the shadow associated with the trapped cell aggregate, which grows over time as more cells are acoustically-trapped, and this is easily identified as the black region identified by the white ellipse in Figure 6Cii–iv. The color adjustment is essentially pulling out the shadow of the sperm pellet, and contrasting it to the background of the channel. This effect is illustrated by Panel Di,ii where two trapping events, beads, and sperm cells with beads, each generate a clearly visible shadow on the bottom of the channel. Following this demonstration of real-time feedback with a mock sexual assault sample, the pellet of sperm was successfully mobilized from the trapping site and captured from the microchip.

## 4. Discussion

The incorporation of this feedback process into the acoustic differential extraction prototype is a significant step towards a more automated, robust system that can handle any type of sexual assault sample. The proof-of-concept data shown in Figure 4, Figure 5 and Figure 6 demonstrate that not only is there a predictable shift in voltage output in response to altering the applied frequency, but also that the optimal trapping frequency will correlate with the lowest voltage output of the piezo. More generally, it is also clear that increasing the viscosity of the solution will cause a shift in the optimal trapping frequency, which can be identified solely by monitoring changes in the minimum output voltage. This shift occurs in controlled samples of glycerol and water, as well as more realistic biofluids like serum and epithelial cell lysate. Figure 6 specifically shows that an initial test with a ‘scanning solution’ is necessary to determine the optimal frequency for each specific chip, but it also shows that the optimal frequency can change based on each specific sample. Most importantly, it shows that by monitoring the output voltage from the piezo, that shift in optimal frequency can be accounted for during the sample trapping, keeping the piezo at its resonant frequency regardless of changes in the liquid environment. This important demonstration has several implications. First, it indicates that the scanning process covering a range of frequencies can be conducted much more rapidly. This is enabled by the ability to measure piezo output voltage during trapping on the millisecond timescale, as opposed to the optical/visual image capture system requiring several seconds with the trapping of fluorescent beads and measure their aggregation. Second, the automated determination of the optimal trapping frequency can be conducted during a trapping of an unknown sample. There is no longer the need to acquire images from each trapping event and quantitate the bead aggregate size at multiple frequencies; in contrast, the local minimum in voltage output can be determined before the scan is even complete. This indicates that if, for example, the optimal trapping frequency is defined early in the frequency scan, a subsequent rise in voltage output solidifies the minimum (optimal frequency) as the previous scan step. At that point the system can abandon the scan and adopt the defined optimal frequency for the remainder of sample trapping. Third, and most important, these findings illustrate that acoustic trapping can be adjusted in real-time to prevent loss of sperm cells during a test. The initial frequency scan provides a starting point for sample trapping, but the ability to scan frequencies during trapping means that no matter what sample is being analyzed, the optimal trapping frequency will be found and implemented every time. In its future automated format, the rapidity of the frequency scan (~100 ms per frequency) combined with the flow rate of the system (45 μL/min) will result in less than 1 nL of sample traversing the trap site during each tested frequency, and the potential for sample loss during the scan is negligible.

In its current form, this feedback system requires manual manipulation and data analysis during acoustic trapping. This approach does effectively trap sperm cells from high E-cell samples, which was formerly problematic [11], but does not yet fully address the overarching problem of the sexual assault backlog. Namely, this technique must be faster and more automated than the current differential extraction protocols. The clear extension of this work is to incorporate automated feedback into the ADE system, by writing a program that will detect the minimum voltage output of the piezo and adjust the optimal trapping frequency independent of any operator. This will provide a form of ‘cruise control’ as the system will be able to adapt to any sample type in real time. Furthermore, the current manual feedback system is limited in terms of speed by the ability of the user to process information and enters in new frequencies at which to trap. When the automated cruise control system is applied, computer processing will conduct an entire scan in hundreds of milliseconds, meaning that near-constant analysis can take place through the sample trapping, ensuring that the optimal trapping frequency is always applied. As a more general future application, it may be feasible to use this technology for the express purpose of sensing material properties in real time. For example, a monitoring rate of cellular lysis via rapid voltage measurements could give precise information about the kinetics of certain reactions, or indicate the degree of cellular rupture.

## 5. Conclusions

Expanding upon excellent work from the Nilsson group, we have applied real time electronic feedback to our novel acoustic differential extraction prototype. By rapidly measuring the voltage output of a piezoelectric transducer and adjusting the applied frequency of sound during acoustic trapping, subtle variations in sample composition can be accounted for to prevent the loss of sperm cells. The novel aspect of this work stems from using different electronic measurements (i.e., voltage instead of impedance), as well as applying the described principles and methods to a range of mock samples and standards. This exciting development means that a broader range of samples will be able to be tested with ADE, and once the feedback system is made automated, no user input will be required.
